# Immune cells drive new immunomodulatory therapies for myocardial infarction: From basic to clinical translation

**DOI:** 10.3389/fimmu.2023.1097295

**Published:** 2023-01-24

**Authors:** Wenjian Nian, Zijian Huang, Cong Fu

**Affiliations:** ^1^ Department of Clinical Medicine, Wannan Medical College, Wuhu, China; ^2^ Department of Cardiology, Yi Ji Shan Hospital affiliated to Wannan Medical College, Wuhu, China; ^3^ Anesthesia Laboratory and Training Center, Wannan Medical College, Wuhu, China; ^4^ Key Laboratory of Non-coding RNA Transformation Research of Anhui Higher Education Institution, Wannan Medical College, Wuhu, China

**Keywords:** myocardial infarction, ventricular remodeling, immune cells, immunomodulatory therapies, translational medicine

## Abstract

The high incidence of heart failure secondary to myocardial infarction (MI) has been difficult to effectively address. MI causes strong aseptic inflammation, and infiltration of different immune cells and changes in the local inflammatory microenvironment play a key regulatory role in ventricular remodeling. Therefore, the possibility of improving the prognosis of MI through targeted immunity has been of interest and importance in MI. However, previously developed immune-targeted therapies have not achieved significant success in clinical trials. Here, we propose that the search for therapeutic targets from different immune cells may be more precise and lead to better clinical translation. Specifically, this review summarizes the role and potential therapeutic targets of various immune cells in ventricular remodeling after MI, especially monocytes/macrophages and neutrophils, as a way to demonstrate the importance and potential of immunomodulatory therapies for MI. In addition, we analyze the reasons for the failure of previous immunomodulatory therapies and the issues that need to be addressed, as well as the prospects and targeting strategies of using immune cells to drive novel immunomodulatory therapies, hoping to advance the development of immunomodulatory therapies by providing evidence and new ideas.

## 1 Introduction

In adult mammals, the substantial loss of cardiomyocytes after MI cannot be compensated by their limited regenerative capacity, ultimately leading to scar formation (1). However, poor ventricular remodeling frequently occurs after MI, which drives the development of heart failure (2). The degree of ventricular remodeling is associated with various pathophysiological processes, but the main determinants are thought to be the initial infarct size and the adequacy of late repair (3). Although in current clinical practice, timely reperfusion can significantly improve infarct size and the effects of the renin-angiotensin-aldosterone system (RAAS) can be blocked by drugs (4), there are still surviving cardiomyocytes that will apoptosis after early reperfusion due to excessive inflammation and the late repair process relies mainly on the body itself to complete the transition from the inflammatory response to the repair response, which is difficult to effective interventions to treat. Therefore, the management of MI may need to focus more on regulating the early inflammatory and late cardiac repair processes.

Cardiac repair after MI can be divided into three phases: the acute inflammatory phase, the fibrous repair phase, and the stabilization phase (5). Early aseptic inflammation contributes to the removal of necrotic tissue and damaged extracellular matrix (ECM), whereas late and timely suppression of inflammation contributes to tissue repair. Thus, dysregulation of various immune pathways, such as excessive early inflammation, inadequate suppression of inflammation during the repair phase, or excessive fibrosis, can lead to poor ventricular remodeling (6, 7).

Various inflammatory factors often directly mediate injurious events, also after MI. However, many previous immunomodulatory therapies targeting inflammatory factors or their corresponding receptors have failed to achieve significant results, suggesting that we need to reconsider the applicability of these targets. Immune cells play the most central role in regulating the balance between inflammation and repair levels during ventricular remodeling, and exploring the role of various immune cells in ventricular remodeling to find more precise targets promises to be a new way to achieve immunomodulatory therapies.

In this review, we focus on the role of monocytes/macrophages, neutrophils, dendritic cells (DCs), and lymphocytes in ventricular remodeling and their potential therapeutic targets, and analyze the current status of immunomodulatory therapies and the prospects and targeting strategies of immune cells as new protagonists of immunomodulatory therapies, aiming to provide evidence for the feasibility and clinical translation of immunomodulatory therapies in MI and new ideas.

## 2 Pathophysiological processes after MI

Necrotic cells and damaged ECM after MI can release damage-associated molecular patterns (DAMPs), including High Mobility Group Protein B1 (HMGB1), S100A8/A9, low-molecular-weight hyaluronic acid, heat shock protein (HSP), ATP, double-stranded DNA, IL-1α, and IL-1β (7, 8). Surviving parenchymal cells and infiltrating various immune cells in the heart express pattern recognition receptors (PRRs) that bind to DAMPs and activate proinflammatory pathways, mainly NF-κB and MAPK pathways, to amplify the inflammatory response through two main processes: (I) promoting the secretion of proinflammatory factors, including TNF-α, IL-1β, IL-6, and IL-18, to further amplify the initial inflammatory response. (II) secretion of CXC- and CC-type chemokines and adhesion molecules, with CXC-type chemokines acting primarily as chemokines for neutrophils in early stages, while CC-type chemokines act primarily as chemokines for monocytes in early stages and also attract T lymphocytes, and adhesion molecules such as VCAM, ICAM, and selectins promote infiltration of circulating leukocytes (9, 10). In addition, there is evidence that mitochondria-rich subcellular membrane components from necrotic cardiomyocytes can trigger complement cascade responses, which can also promote upregulation of chemokine levels after MI (11, 12). Thus, the release of DAMPs can mediate the dynamic recruitment of immune cells, leading to a massive recruitment of monocytes and neutrophils during the inflammatory phase. A fraction of monocytes infiltrating the injury site can differentiate into macrophages according to the microenvironment, and all three together phagocytose necrotic cells and damaged ECM, amplifying the inflammatory response (7).

Thereafter, timely suppression of inflammation and clearance of apoptotic neutrophils can facilitate the onset of the repair response and enter the fibrous repair phase (5, 13). During this period, DCs and lymphocyte infiltration increases and the monocyte/macrophage phenotype changes to a repair phenotype, secreting cytokines such as TGF-β, VEGF, and IL-10, which support inflammation regression, vascular regeneration, and scar formation (14–16).

**Figure 1:**
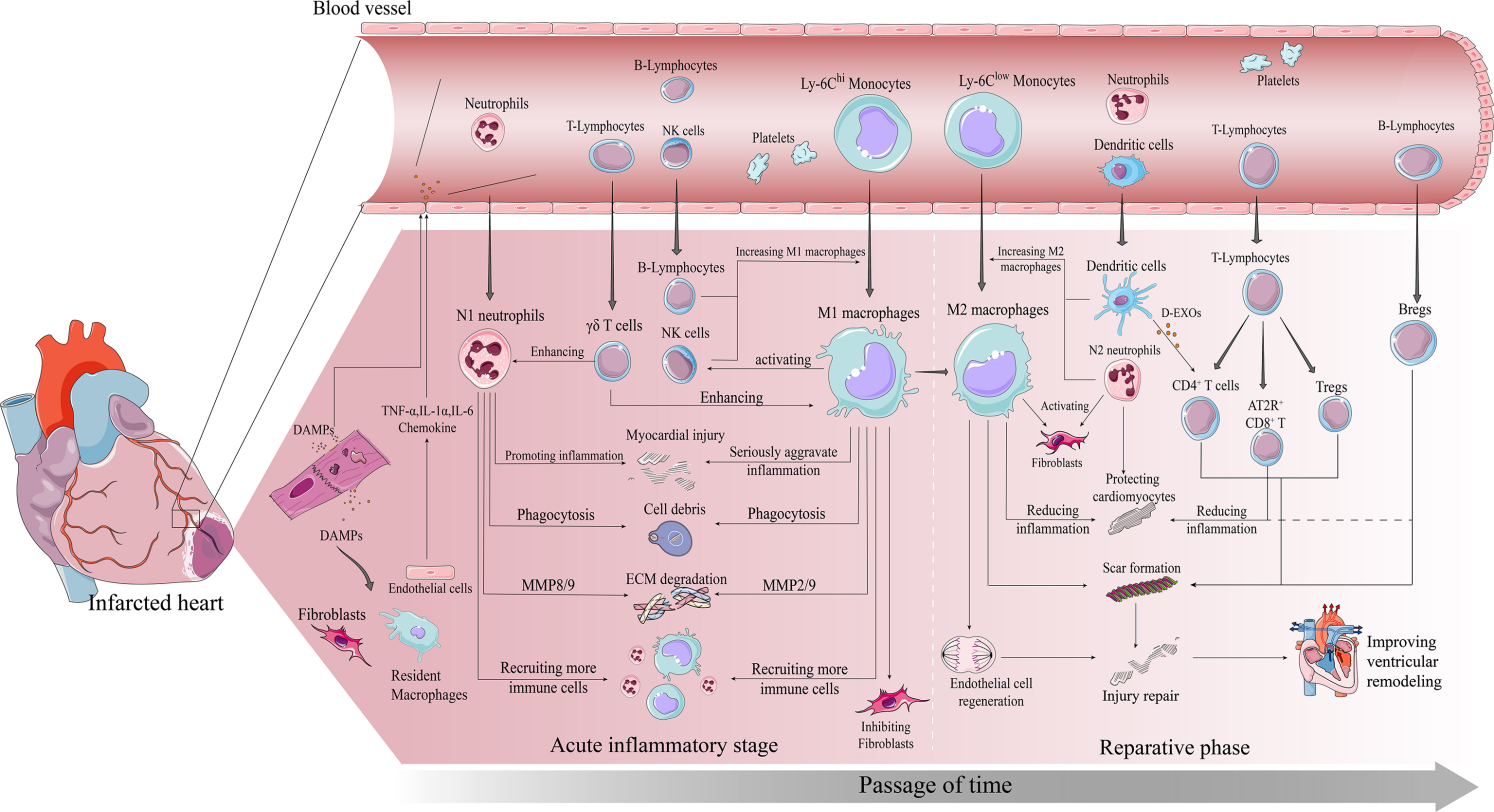
Immune cascade after MI. early post-MI necrotic cells release DAMPs which bind to surviving parenchymal cells releasing pro-inflammatory and chemokines and recruiting immune cells to infiltrate. The inflammatory phase is dominated by infiltration of Ly-6C^hi^ monocytes, M1 macrophages and neutrophils. All three work together to degrade the ECM, phagocytose necrotic cells, and amplify the inflammatory response to recruit more pro-inflammatory immune cells. The repair phase focuses on resolution of inflammation and cardiac repair, with Ly-6C^low^ monocytes, M2 macrophages, and Tregs infiltrating predominantly. All three work together to suppress inflammation, promote fibroblast activation involved in scar formation, and promote vascular regeneration, ultimately preventing further injury and improving ventricular remodeling in time.

The stabilization phase mainly occurs with extracellular matrix cross-linking, repair cell inactivation or apoptosis, and myocardial fibroblast quiescence in the infarct area, leading to the maturation and stabilization of scar tissue and marking the end of the MI repair process *in vivo* (7, 17) (**Figure 1**).

Thus, various immune cells play an extremely important and complex role in ventricular remodeling and play a key regulatory role in the smooth transition from early inflammation to late repair, as we will describe in detail below.

## 3 Powerful modulation of ventricular remodeling by immune cells

**Figure 2:**
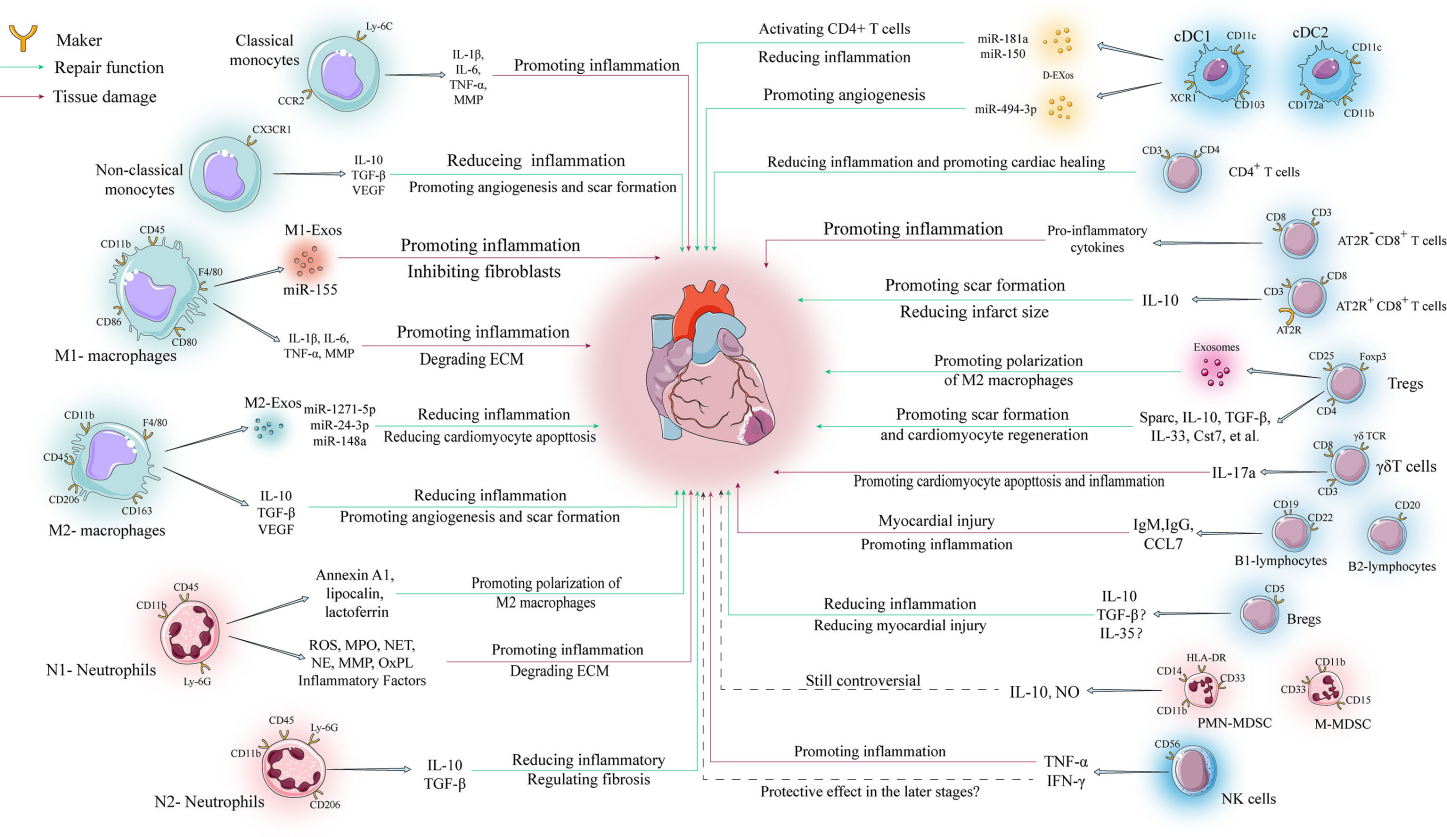
Regulatory mechanisms of various immune cells in ventricular remodeling. The involvement of immune cells in ventricular remodeling is often bidirectional, with various immune cells secreting different cytokines to perform different functions depending on their phenotype during different stages of ventricular remodeling.

Here, we summarize the regulatory mechanisms and some potential targets played by monocytes/macrophages, neutrophils, DCs, T cells, B cells, myeloid-derived suppressor cells (MDSCs), and natural killer cells (NK cells) on ventricular remodeling (**Figure 2** and **Table 1**).

**Table 1:** Characteristics, functions, and potential targets of various immune cells involved in post-myocardial infarction.

Cell type	Subtype	Maker	Peak infiltration	Active molecule	Biological effect	Potential Target
Monocytes	Classical monocytes	Ly-6C^hi^ CCR2^+^ CX3CR1^low^	Day 5 after MI	IL-1β, IL-6, TNF-α, MMP	Promoting inflammatory environment Engulfing necrotic tissue	/
	Non-classical monocytes	Ly-6C^low^ CCR2^-^ CX3CR1^hi^	Day 7 after MI	VEGF, IL-10, TGF-β	Promoting angiogenesis, scar formation and inflammation resolution	Ep3
Macrophages	M1- macrophages	CD11b^+^ CD45^+^ F4/80^+^ CD80^+^ CD86^+^	Day 5 after MI	IL-1β, IL-6, TNF-α, MMP, iNOS, IL-23, Exosomes	Powerful phagocytosis and pro-inflammatory ability	Lgr4, Dectin-1, miR-155
	M2- macrophages	CD11b^+^ CD45^+^ F4/80^+^ CD163^+^CD206^+^	Day 7 after MI	VEGF, IL-10, TGF-β, Arg-1, Exosomes	Powerful reparative and anti-inflammatory abilities	NR4A1, IRF-5, miR-148a miR-24-3p, miR-1271-5p
Neutrophils	N1- Neutrophils	CD11b^+^ CD45^+^ Ly-6G^+^ CD206^-^	Day 1 after MI	ROS, MPO, NET, NE, MMP, Pro-inflammatory cytokines annexin A1, lipocalin, lactoferrin	Promoting inflammatory environment Engulfing necrotic tissue Promoting polarization of M2 macrophages	OxPL
	N2- Neutrophils	CD45^+^ CD11b^+^ Ly-6G^+^ CD206^+^	/	IL-10, TGF-β	Regulating fibrosis and protecting heart function	/
DCs	cDCs	cDC1:CD11c^+^ CD103^+^ XCR1^+^	Day 7 after MI	Exosomes	Activating CD4^+^ T cells and CD8^+^ T cells The specific effects on ventricular remodeling need to be further elucidated depending on the different subtypes	miR-494-3p, IRAK-4, miR-181a, miR-150
		cDC2:CD11c^+^ CD11b^+^ CD172a^+^				
	pDCs	CD11c^+^ CD123^+^ BDCA2^+^	/	IFN-1	No significant effect on cardiac function	/
T cells	CD4^+^ T cells	CD3^+^ CD4^+^	Day 7 after MI	/	Reducing the inflammatory response Promoting cardiac healing	/
	CD8^+^ T cells	CD3^+^ CD8^+^ AT2R^-^	Day 7 after MI	Pro-inflammatory cytokines	Promoting inflammatory response	AT2R
		CD3^+^ CD8^+^ AT2R^+^	Day 7 after MI	IL-10	Promoting scar formation Reducing infarct size	
	Tregs	CD4^+^ CD25^+^ Foxp3^+^	Day 7 after MI	Sparc, IL-10, TGF-β, IL-33, Cst7, TNFSF11, FGL2, MATN2, IGF2, Exosomes	Reducing the inflammatory response Promoting scar formation Stimulating cardiomyocyte regeneration Promoting polarization of M2 macrophages	Sparc, mTORC1, CCL17
	γδT	CD3^+^ CD8^+^, γδ TCR	Day 7 after MI	IL-17a	Promoting inflammatory and cardiomyocyte death	/
B cells	B1-lymphocytes	CD19^+^ CD22^+^	Day 7 after MI	IgM, IgG, CCL7	Antibody deposition Promoting inflammation and myocardial injury	miR-21/HIF-1α, BAFF
	B2-lymphocytes	CD20^+^				
	Bregs	CD5^+^	/	IL-10, TGF-β?IL-35?	Reducing inflammation and myocardial injury	/
MDSCs	M-MDSCs	CD11b^+^ CD14^-^ CD15+ HLA-DR^-^ CD33^+^	/	IL-10, NO	Whether MDSCs can protect cardiomyocytes is still controversial and needs further investigation	AAR
	PMN-MDSCs	CD11b^+^ CD14^+^ CD15^-^ HLA-DR^low^ CD33^+^				
NK cell	/	CD56^+^	Day 7 after MI	TNF-α, IFN-γ	Pro-inflammatory effect Protective effect in the later stages?	/

### 3.1 Monocytes/macrophages

The role of monocytes and macrophages after MI has been the most extensively studied to date, and a large body of evidence suggests that monocytes/macrophages play a central role in the regulation of inflammation after MI.

The adult mouse heart contains two major populations of resident macrophages: CCR2^-^ MHC-II^low^ and CCR2^-^ MHC-II^hi^, which account for approximately 7-8% of all non-cardiomyocytes, are predominantly derived from the yolk sac or fetal liver, and provide immune monitoring and regulation of cardiac function (18). In the physiological state, resident macrophage populations may proliferate to maintain relative stability in number and function, but they are rapidly depleted after MI and thereafter rely primarily on recruitment of circulating monocytes that then differentiate into macrophages to regulate the development and resolution of inflammation (18, 19).

Significant monocyte mobilization occurs early after MI, and even though recruitment starts first from the vascular pool, the main provider remains the spleen (20, 21). During the inflammatory phase, CCL-2/MCP-1 recruits Ly-6C^hi^ CCR2^+^ CX3CR1^low^ monocytes, also known as classical monocytes, by binding CCR2, which have distinct pro-inflammatory properties and can differentiate into M1-type macrophages (CD11b^+^ CD45^+^ F4/80^+^ CD80^+^ CD86^+^); in contrast, during the repair phase, Ly-6C^low^ CCR2^-^ CX3CR1^hi^ monocytes, also known as non-classical monocytes, rely mainly on CXC3R1 recruitment, have anti-inflammatory properties and can differentiate into M2-type macrophages (CD11b^+^ CD45^+^ F4/80^+^ CD163^+^ CD206^+^) (22, 23).

The role of monocytes and macrophages after MI is bidirectional. It has been demonstrated that the inflammatory phase is characterized by the infiltration of large numbers of Ly-6C^hi^ monocytes and M1-type macrophages into the heart, with their numbers peaking on day 5 (24). They cooperate with recruited neutrophils to secrete inflammatory substances such as IL-1β, IL-6 and TNF-α in response to increased DAMPs, exacerbating the proinflammatory environment, and secrete large amounts of matrix metalloproteinases (MMPs), especially MMP-2 and MMP-9, which are particularly important for degrading the damaged ECM and setting the stage for later repair processes (25, 26). In contrast, Ly-6C^low^ monocytes and M2-type macrophages infiltrate mainly during the repair phase and they are considered as anti-inflammatory cells, both peaking at day 7 post-infarction (24), secreting mainly TGF-β, IL-10 and VEGF, mediating collagen production, myofibroblast activation and vascular regeneration (27, 28). Thus, M2-type macrophages mainly mediate the transition from inflammation to fibrosis during the repair phase. Under the influence of M2-type macrophages, myocardial fibroblasts can activate, proliferate, and migrate to the infarct zone to produce collagen I and collagen III that are involved in scar formation (14, 29). Furthermore, surprisingly, recent evidence suggests that macrophages have the ability to transition to a fibroblast phenotype, which brings new ideas to regulate the degree of fibrosis after MI (30).

In conclusion, the regulatory role of monocytes/macrophages is present throughout the process of ventricular remodeling, so therapeutic approaches targeting monocytes/macrophages have great potential. However, previous studies have shown that a large increase in macrophage numbers after infarction leads to excessive inflammation and impairment of cardiac function, while depletion of infiltrating macrophages significantly impairs wound healing and increases poor ventricular remodeling and mortality (31, 32). These results suggest that achieving a balance between inflammation and fibrosis *via* monocytes/macrophages requires finding more precise targets.

Therefore, in recent years, monocyte/macrophage-based research has focused more on two directions: (I) Searching for targets that inhibit inflammation during the inflammatory phase or promote the repair response during the repair phase. Tang et al. demonstrated that inhibition of the Ep3 receptor in mice blocked the recruitment of Ly-6C^low^ monocytes by the PGE2/Ep3 axis and also inhibited TGF-β1 signaling and VEGF secretion, which was detrimental to vascular regeneration and scar formation, suggesting that Ep3 plays a role in promoting cardiac repair has an important role (33). Huang et al. found that cardiac function and cardiac remodeling were significantly improved in macrophage-specific Lgr4 knockout mice, and that Lgr4 deletion reduced infiltration of Ly-6C^hi^ monocytes and M1 macrophages, thereby reducing the inflammatory response in infarcted myocardial tissue (34). Fan et al. found that macrophage-specific Dectin-1 deletion resulted in reduced CXCL1 and G-CSF expression and therefore reduced infiltration of Ly-6C^hi^ monocytes and neutrophils, leading to a significant improvement in cardiac function and reduced apoptosis of cardiomyocytes (35). (II) Searching for targets that regulate macrophage polarization. It was shown that Nuclear Receptor Subfamily 4 Group A Member 1 (NR4A1) plays an important role in the polarization of macrophages to the M2 phenotype, and the deficiency of NR4A1 leads to overexpression of IL-6, TNF-α, IL-6, IL-1, and MMP-9, which inhibits the conversion of macrophages to the M2 phenotype and leads to poor cardiac healing (36). Another study showed that if interferon regulatory factor-5 (IRF5) was silenced *in vivo*, it could reduce the polarization of M1 macrophages after MI, resulting in more efficient resolution of inflammation and less adverse ventricular remodeling (37).

In addition, it is worth mentioning that exosomes have received much attention in recent years as an important mode of intercellular communication. Recent evidence suggests that the regulation of inflammation by macrophages after MI is also associated with exosomes. M1 macrophage-derived exosomes (M1-exos) highly express various pro-inflammatory miRNAs that impair endothelial cell angiogenesis and increase cardiac rupture and inflammatory responses (38). More importantly, miR-155 delivered by M1-exos not only exacerbated the inflammatory response but also inhibited fibroblast activity, thereby exacerbating the risk of cardiac rupture, a result that suggests the therapeutic potential of our targeted inhibition of miR-155 (39). In contrast, M2 macrophage-derived exosomes (M2-exos) delivered miR-1271-5p, miR-148a, and miR-24-3p to cardiomyocytes to attenuate inflammation and cardiomyocyte apoptosis, thereby protecting cardiac function (40–42). These results suggest that macrophage-derived exosomes are extensively involved in the pathophysiological process of ventricular remodeling and the regulation of cellular communication networks between macrophages and other cells. Focusing on the communication between macrophages and other cells, especially through exosomes to identify key communication cargoes for targeted therapies, is also a feasible approach (43).

In conclusion, monocytes/macrophages play a central role in the regulation of inflammation in ventricular remodeling, and how to balance the level of inflammation in monocytes/macrophages is important to prevent adverse ventricular remodeling. Therefore, future studies targeting monocytes/macrophages could be combined with gene sequencing to screen for differentially expressed genes after MI and further explore more precise targets. Furthermore, although differentiating macrophages by M1 and M2 facilitates our studies and interventions, this does not fully reflect the variability between different macrophages, and more detailed transcriptomic and metabolomic analyses of macrophages should be performed at the single-cell level to further refine macrophage subpopulation and function.

### 3.2 Neutrophils

Neutrophils are usually defined as CD11b^+^ CD45^+^ Ly-6G^+^, and in MI, neutrophils have been shown to be the first cell lineage to be recruited to the heart (24). CXCL1/2-CXCR2 signaling activation is the main pathway for neutrophil recruitment (44), and in addition, HMGB1 can form a heterodimer with CXCL12 and binding to CXCR4 can also induce neutrophil recruitment and migration (45).

As previously described, neutrophils recognize DAMPs and chemokines and release large amounts of pro-inflammatory factors to amplify the local inflammatory response. In addition, activated neutrophils release large amounts of ROS, proteases, and neutrophil extracellular traps (NETs), creating a continuous pro-inflammatory environment (46, 47). The major proteases released by neutrophils include myeloperoxidase (MPO), neutrophil elastase (NE), MMP-8 and MMP-9, which are the main substances for neutrophils to perform their functions.

MPO is the main functional protein of neutrophils (48). Studies have shown that MPO knockdown attenuates pathological cardiac remodeling and that inhibition of MPO also reduces inflammation and decreases left ventricular dilation and dysfunction (49, 50). NE is a serine protease, and plasma NE concentrations are significantly elevated in patients with MI (51). NE degrades collagen and fibrinogen and impairs myocardial contractility after MI (52). Interestingly, in the hearts of NE knockout mice, insulin receptor 1 expression levels and Akt phosphorylation were significantly upregulated, suggesting that NE deficiency may improve ventricular remodeling by upregulating insulin/Akt signaling (53). the MMP family primarily mediates ECM degradation, and activated MMP-8 effectively degrades collagen, which may be associated with poor ventricular remodeling early after MI. However, a study showed that upregulation of MMP-8 levels in patients with advanced MI positively correlated with LV function, suggesting that the role of MMP-8 may be bidirectional (54). MMP-9 is one of the most widely studied members of the MMP family, and neutrophils are the most prominent early source of MMP-9 in MI (55). MMP-9 knockdown enhances the expression of multiple anti-inflammatory genes to promote polarization of M2 macrophages (56). Furthermore, in ischemia-reperfusion injury, left ventricular infarct size was reduced by 35.4% in MMP-9 knockout mice (57). Thus, the current evidence suggests that inhibition of MMP-9 expression attenuates the inflammatory response and improves ventricular remodeling. NETs consist of depolymerized chromatin and intracellular granule proteins and are produced by activated neutrophils through a new form of death called NETosis, which is distinct from apoptosis and cell necrosis (58). In addition, NETosis allows neutrophils to abundantly release of S100A8/A9 proteins and induce the recruitment of more neutrophils (59). NETs can activate macrophage NLRP3 to produce IL-1β and IL-18, thus exerting powerful proinflammatory effects, where IL-18 in turn can promote an increase in circulating NETs, creating a vicious cycle that further amplifies inflammation (60, 61). Here, notably, patients with common LNK loss-of-function activate oxidized phospholipid (OxPL)-dependent platelet-neutrophil aggregation *in vivo*, promoting NETosis (62), and it has been demonstrated that inactivation of OxPL by antibodies after MI in mice protects cardiomyocyte viability and reduces infarct size (63). Therefore, the role of OxPL in the development of cardiovascular disease deserves further investigation and neutrophils may play an important role as mediators. Furthermore, some studies focusing on neutrophil-derived extracellular vesicles have found that they enhance endothelial microvascular permeability under inflammatory conditions and can promote endothelial cell production of inflammatory factors, exacerbating inflammation (64).

Thus, it was previously thought that neutrophils primarily mediate destructive effects. In fact, neutrophil recruitment is necessary to allow a shift from an inflammatory to a reparative response after MI. Studies have shown that apoptotic neutrophils inhibit continued neutrophil recruitment, stimulate macrophages to engulf apoptotic neutrophils, and cause macrophage polarization to the M2 phenotype by secreting annexin A1, lipocalin, lactoferrin, and apoptotic vesicles (65). Furthermore, another study showed that neutrophils induce upregulation of TGF-β1 expression in fibroblasts, thereby promoting the resolution of inflammation, which is essential for late scar formation, again demonstrating the need for the presence of neutrophils (66). More importantly, today’s evidence allows the division of neutrophils into two subpopulations: N1 type and N2 type. N1 type neutrophils are defined as Ly-6G^+^ CD206^-^ and N2 type neutrophils are defined as Ly-6G^+^ CD206^+^, similar to M1 and M2 macrophages (67). IL-10, TGF-β and lipid mediators from N2 type neutrophils have been shown to exert anti-inflammatory effects, again suggesting a regulatory role of neutrophils on fibroblasts (13). In addition, histamine from neutrophils reduces microcirculatory thrombosis and vascular inflammation in MI hearts, and also improves cardiac function and fibrosis by reducing ROS and NETs *in vivo* (68, 69). In conclusion, there is growing evidence that neutrophils may produce different outcomes due to phenotypic alterations, a finding that could explain why clinical trials of neutrophil inhibition have not been successful (70, 71) (**Table 2**).

**Table 2:** Results of several major clinical studies based on immunomodulatory protection against myocardial ischemia.

Targets	Drugs	Patient population	Outcome	Ref
Neutrophils	rhuMAb CD18	394 patients with STEMI who underwent thrombolysis	No significant effect on MI size	(70)
Neutrophils	Hu23F2G, a humanized MAb to CD11/CD18	420 patients with STEMI treated with PCI	No significant effect on MI size	(71)
TNF-α	Infliximab	150 patients with stable New York Heart Association class III or IV heart failure and left ventricular ejection fraction <or=35%	Neither the low dose (5 mg/kg) nor the high dose (10 mg/kg) improved the clinical status of patients through 14 weeks, but patients receiving the high dose had an increased risk of death or hospitalization for heart failure through 28 weeks.	(72)
IL-1R	Anakinra	25 patients with STEMI treated with PCI	No significant improvement in left ventricular function within 14 days	(73)
	Anakinra	182 patients with NSTEMI undergoing PCI	hsCRP was significantly decreased, but MACE (death, stroke, recurrent MI) was increased	(74)
IL-1β	Canakinumab	10,061 patients with previous MI and a hsCRP level of 2 mg or more per liter	Decreased incidence of MACE but increased incidence of neutropenia and fatal infections	(75)
IL-6	Tocilizumab	117 patients with NSTEMI undergoing PCI	Effectively reduced perioperative myocardial injury	(76)
Complement cascade C5	Pexelizumab	960 patients with STEMI treated with PCI	No significant effect on MI size and associated clinical events	(77)
	Pexelizumab	3099 patients treated with CABG	No significant effect on MI size and associated clinical events	(78)
Complement cascade C1	C1 esterase inhibitor (C1-INH)	80 patients with STEMI undergoing CABG	Reduced perioperative myocardial injury	(79)
P-selectin	Inclacumab	322 patients with NSTEMI undergoing PCI	Effectively reduced postoperative myocardial injury	(80)

As the beneficial functions of neutrophils are gradually being tapped, more attention has been paid to how to target neutrophils to promote the resolution of inflammation. Interestingly, scholars have developed engineered neutrophil apoptotic bodies that mimic the role of apoptotic neutrophils in promoting the polarization of M2 macrophages, showing high efficacy in a rat MI model (81). Similarly, in another study, neutrophil-mimetic liposomes (Neu-LPs) were created by fusing neutrophil membranes with liposomes, which inherit neutrophil surface antigens but without neutrophil activity. Neu-LPs target the infarcted heart, neutralize pro-inflammatory cytokines, thereby suppressing intense inflammation and modulating the immune microenvironment (82). Furthermore, in a mouse MI model, administration of PF-1355 (MPO inhibitor) for 7 days reduced inflammatory cell infiltration and attenuated left ventricular dilation, and MPO inhibition has been shown to improve ischemia-related cardiac remodeling in animal experiments (50).

In conclusion, the studies prompting a balance between pro- and anti-inflammatory neutrophils remain imperfect, and in the future, how to target the shift in neutrophil phenotype may be more beneficial than treatment with anti-inflammatory strategies alone.

### 3.3 DCs

DCs are the most powerful antigen-presenting cells, activating T lymphocytes and secreting various cytokines to maintain immune homeostasis. DCs are usually divided into 2 subpopulations. Conventional DCs (cDCs) and plasmacytoid DCs (pDCs). cDCs can be further divided into cDC1 (CD11c^+^ CD103^+^ XCR1^+^) and cDC2 (CD11c^+^ CD11b^+^ CD172a^+^). pDCs are defined as CD11c^+^ CD123^+^ BDCA2^+^ and their main function is to secrete IFN-1 to inhibit viruses and bacteria (83). Previous studies have shown that bone marrow-derived DCs peak on the seventh day post-infarction (24).

Anzac et al. found that the absence of CD11c^+^ DCs in the bone marrow of mice increased the expression of pro-inflammatory factors after MI and promoted the infiltration of Ly-6C^hi^ monocytes and M1 macrophages, leading to poor ventricular remodeling (84). Another study found that a decrease in the number of DCs in human infarcted myocardial tissue was also associated with an increase in macrophage infiltration, leading to poor cardiac repair (85). Furthermore, DC-derived exosomes (DEXs) have been shown to promote the entry of CD4^+^ T cells into the infarct zone and the delivery of miR-494-3p to endothelial cells to promote vascular regeneration, thereby protecting cardiac function (86, 87). These results suggest that DCs can attenuate the inflammatory response and poor ventricular remodeling in MI. However, studies on different subtypes of DCs reported different results. Lee et al. found that depletion of CD11b^+^ and CD103^+^ cDCs in mice attenuated the inflammatory response after MI and thus improved cardiac function, whereas specific elimination of pDCs in mice had no significant effect on cardiac function (88). However, in a myocardial ischemia-reperfusion model, IFN-1 secreted by pDCs instead exacerbated reperfusion injury, whereas removal of pDCs led to a reduction in infarct size (89). These results suggest that cDCs may also have different effects depending on phenotypic differences, and the role of pDCs on MI needs further elucidation.

Available evidence is more supportive of the overall beneficial effects of DCs on ventricular remodeling, and several preclinical studies targeting DCs have shown some therapeutic potential. Maekawa et al. reported that deletion of IL-1 receptor-associated kinase 4 (IRAK-4) attenuated the mobilization of deleterious DCs to attenuate the inflammatory process (90). Another study showed that miR-181a and miR-150 could protect cardiomyocytes by attenuating the immune inflammatory response of DCs through JAK1-STAT1/c-Fos signaling (91). Interestingly, a study combining DEXs with alginate hydrogels to form DEXs-Gel found that DEXs-Gel maintained the release of DEXs and prolonged the retention of DEXs without adverse effects on migration *in vivo*. In addition, DEXs-Gel better activated regulatory T cells and shifted macrophages to M2 macrophages (92). However, while rejoicing in these results, we must be aware that the immune mechanisms of DCs after MI remain unclear, which may limit the development of clinical trials, and continued in-depth exploration of the functions of different subpopulations of DCs remains to be addressed.

### 3.4 T cells

T cells are usually divided into cytotoxic T cells (CD8^+^) and helper T cells (CD4^+^). In addition, there are two specific subsets of T cells, one called regulatory T cells (Tregs), which highly express the Forkhead box protein P3 (Foxp3) and suppress inflammatory responses, and the other called γδ T cells, whose TCRs are composed of γ and δ chains and secrete a variety of cytokines and chemokines that are involved in immune regulation (93).

It has been demonstrated that all four of these T cell subsets are involved in the ventricular remodeling process, with numbers all peaking at day 7 post-MI (24). It was shown that RAG1 knockout mice had a smaller infarct size after MI due to the lack of T cells (94). However, for CD4^+^ T cells, Hofmann et al. found that the lack of CD4^+^ T cells increased the infiltration of Ly-6C^hi^ monocytes in the infarct area, which exacerbated the inflammatory response and led to poor cardiac healing (95). In addition, a decrease in Th2 lymphocytes was associated with MI patients with a high risk of adverse cardiovascular events (96). These results suggest that CD4^+^ T cells may play a protective role after MI. For CD8^+^ T cells, a study showed that mice lacking CD8^+^ T cells had improved cardiac function and lower mortality in the early post-MI period, but in the later period resulted in poor scar formation and an increased risk of cardiac rupture (97). Corroborating this, circulating CD8^+^ T cell levels in MI patients were positively correlated with cardiovascular mortality in the short term (98). However, in the later period, Curato et al. reported that AT2R^+^ CD8^+^ T cells significantly accumulate on day 7 after MI and are able to produce IL-10 in response to angiotensin II stimulation, promoting scar formation. Furthermore, unlike classical AT2R^-^ CD8^+^ T cells, transplantation of AT2R^+^ CD8^+^ T cells into the heart significantly reduced infarct volume (99). Thus, CD8^+^ T cells may cause cardiac injury in the early stages but exert a reparative effect at a later stage due to AT2R expression. In conclusion, these results reflect the complexity of the role of T cells after MI; however, most importantly, it is now established that the most important implementers of the protective effect of T cells on MI are Tregs.

It was shown that Tregs were highly enriched after myocardial injury, mainly from the thymus, with a smaller contribution from conventional CD4^+^ T cell differentiation. Furthermore, RNA sequencing showed that Tregs infiltrating after myocardial injury had a large number of differentially expressed transcripts compared with normal conditions, exhibiting a repair-promoting phenotype, with a highly expressed secreted protein acidic and rich in cysteine (Sparc) increasing collagen production and potentially acting as a key target for cardioprotection (100). Corroborating this, in mice lacking Tregs, the inflammatory response after MI was exacerbated, whereas transplantation of Tregs significantly reduced inflammation and prevented adverse ventricular remodeling, one reason being that Tregs promote M2 macrophage polarization (101–103). Interestingly, the paracrine role of Tregs also plays a protective role, starting with Treg-derived exosomes that also promote M2 macrophage polarization and secondly Tregs secretes six major factors (Cst7, Tnfsf11, Il33, Fgl2, Matn2 and Igf2) to stimulate cardiomyocyte proliferation (104, 105). In conclusion, these results respond to the importance of Tregs for myocardial injury repair.

For γδ T cells, Yan et al. found that IL-23 secreted by M1 macrophages and neutrophils after MI drove cardiac γδ T cells to produce IL-17a, which in turn exacerbated cardiomyocyte injury by promoting infiltration of neutrophils and macrophages, creating a vicious cycle. Furthermore, lack of IL-23, IL-17a or γδ T cells improved cardiac function and survival after MI in mice (106). These results suggest that γδ T cells play a predominantly pro-inflammatory role after MI.

Therapies targeting T cells currently focus on Tregs, as they exert a powerful pro-repair capacity. Yang et al. demonstrated that activation of mTORC1 promotes Tregs activation to suppress macrophage inflammation, suggesting that targeting the mTORC1 signaling pathway to Tregs has therapeutic implications (107). Feng et al. found that CCL17 competitively inhibits CCL22-stimulated ARRB signaling and Tregs migration, impairing the cardioprotective effects of Tregs, suggesting that inhibition of CCL17 may be an effective strategy to promote Tregs recruitment and suppress myocardial inflammation (108). Notably, chimeric antigen receptor (CAR) -T cell therapies were first used to treat cardiac fibrosis in mice by Aghajanian et al. with significant results (109). However, they can be off-target and have organ-specific toxicity, and minimizing off-target effects is a problem to be solved.

### 3.5 B cells

The main function of B cells is to produce antibodies and participate in the humoral immune response. b cells can be divided into type B1, type B2 and regulatory B cells (Bregs). type B1 cells highly express CD19 and CD22, type B2 cells highly express CD20 and Bregs cells highly express CD5 (110). After MI, the number of B cells infiltrating the infarcted heart continues to increase, reaching a peak at day 7 (24).

Previous studies have shown that specific depletion of B cells by CD20 monoclonal antibodies or knockdown of B-cell activating factor (BAFF) can improve cardiac function by reducing infarct size and inflammatory response. Importantly, one mechanism is that B cells can secrete CCL7 and exacerbate myocardial injury by recruiting Ly-6C^hi^ monocytes through the CCL7/CCR2 axis (111). This is supported by a recent study in which Sun et al. suggested that B cells recruited after MI can exacerbate myocardial injury by mediating CCL7 secretion through the miR-21/HIF-1α pathway and that B cell-specific deletion of miR-21 and HIF-1α improves cardiac function (112). In addition, blockade of IgM significantly reduced myocardial ischemia-reperfusion injury, suggesting that antibodies produced by B cells may also mediate the effects of myocardial injury after MI (113).

However, B cells also have beneficial effects on ventricular remodeling. intra-myocardial injection of B cells after MI has been shown to reduce apoptosis and improve cardiac function, although this effect may be achieved mainly through the paracrine pathway (114, 115). In addition, a recent study showed that post-MI transplantation of Bregs reduced infiltration of Ly-6C^hi^ monocytes, thereby reducing myocardial injury. Interestingly, the mechanism involved is that Bregs reduce monocyte CCR2 expression by secreting IL-10, whereas IL-10 antibody treatment eliminates this protective effect (116). However, previous studies of Bregs have shown that in addition to IL-10, Bregs function is dependent on TGF-β and IL-35 (117), and IL-35 has been shown to be beneficial for ventricular remodeling after MI (118), yet whether Bregs acts through TGF-β and IL-35 after MI needs to be further explored.

### 3.6 Myeloid-derived suppressor cells

In mice, MDSCs are usually labelled as CD11b^+^ Gr-1^+^. More precisely, MDSCs can be further classified as M-MDSC (CD11b^+^ CD14^-^ CD15^+^ HLA-DR^-^ CD33^+^) and PMN-MDSC (CD11b^+^ CD14^+^ CD15^-^ HLA-DR^low^ CD33^+^) (119).

MDSCs were previously thought to play an important tumor-protective role in the tumor microenvironment (120), but our previous study found that splenic-derived MDSCs were heavily recruited and exacerbated myocardial injury within 24 hours after MI, and blockade of the A adenosine receptor (AAR) inhibited MDSCs mobilization, thereby improving cardiac systolic function (121). Thus, AAR-mediated mobilization of MDSCs may be a potential therapeutic target after MI, however, the exact mechanism remains to be elucidated. Interestingly, MDSCs have also been shown to play a cardioprotective role; Zhou et al. reported that MDSCs could exert anti-inflammatory effects on cardiomyocytes through secretion of IL-10 and NO, thereby alleviating heart failure (122). Another study showed that exercise training stimulated macrophages to induce MDSCs proliferation through IL-10/STAT3/S100A9 signaling pathway, and the increase in MDSCs significantly improved heart failure symptoms (123).

In conclusion, the role of MDSCs in MI remains controversial, which may not be surprising given the complexity of the MDSC phenotype, and it should be determined first which phenotype mediates the damaging or protective effect exerted by MDSCs.

### 3.7 Natural killer cell

Human mature NK cells express the transcription factor T-bet and produce IFN-γ, perforin and granzyme B and can be divided into two subpopulations: CD3^-^ CD56^dim^ and CD3^-^ CD56^hi^ (124). The number of NK cells infiltrating the heart continues to increase after MI and reaches a peak on day 7 (24).

It was shown that patients with MI have an upregulated proportion of circulating NK cells and produce large amounts of TNF-α and IFN-γ (125), in addition, patients with acute coronary syndrome have fewer NK cells in the peripheral blood compared to patients with stable angina (126), implying that NK cells may intervene early in the acute inflammatory response.

Current preclinical studies of NK cells have not clearly elucidated the role they play after MI. It has been reported that NK cell-derived IFN-γ promotes the differentiation of mouse monocytes towards inflammatory DCs and M1 macrophages, and that activated DCs and macrophages co-secrete IL-12 and IL-18, which promote NK cell proliferation, creating a vicious cycle that amplifies the inflammatory response (127). However, it has also been reported that NK cell deficiency reduces apoptosis in cardiac myocytes and that NK cells can inhibit collagen production by cardiac fibroblasts and limit cardiac fibrosis (128, 129).

The contrary results presented so far suggest that NK cells in MI may intervene early in the inflammatory response and continue to function, and may play a beneficial role in ventricular remodeling at a later stage, however more mechanisms remain to be explored.

## 4 Immunomodulatory therapy for MI

As the powerful regulatory roles played by various inflammatory factors and immune cells in ventricular remodeling continue to be elucidated, the development of new targets from the immune inflammatory process after MI to improve ventricular remodeling and to drive the birth of immunomodulatory therapies for MI are promising avenues for clinical translation. For MI, immunomodulatory therapies targeting the control of the degree of inflammation and fibrosis during ventricular remodeling are important for the prevention of adverse ventricular remodeling and distant heart failure, and deserve our attention and consideration.

However, based on the results of existing preclinical trials, clinical studies conducted in recent years have focused more on some inflammatory factors and related receptors, and have not achieved satisfactory results. Here, we summarize the results of several major clinical trials for illustrative purposes (**Table 2**).

### 4.1 Targeting inflammatory factors and related receptors

Inflammatory factors and related receptors are easier to target, and as mentioned earlier, TNF-α, IL-1β, and IL-6 mediate strong inflammatory and degrading effects early and are detrimental to late ventricular remodeling; however, early blockade has not achieved significant results.

For TNF-α, Eugene et al. conducted a clinical trial with infliximab (a chimeric monoclonal antibody to TNF-α) in patients with heart failure and found that short-term application of infliximab did not improve clinical symptoms and that high doses were detrimental to the long-term prognosis of patients (72).

For IL-1β, the results from a clinical trial of recombinant IL-1R antagonist (anakinra) found lower rates of final mortality or hospitalisation for heart failure in STEMI patients taking anakinra compared with placebo, with no significant difference in the rate of serious infections. However, the small sample size of this trial led to results that require further validation (73). For NSTEMI patients, anakinra led to an overall increase in major adverse cardiovascular events (MACE) in patients with NSTEMI, despite a significant reduction in high-sensitivity C-reactive protein (hsCRP) (74). In another trial, specific blockade of IL-1β with canakinumab prevented MACE over a median of 3.7 years. however, canakinumab caused a significantly increased probability of fatal infection compared with the placebo group and was not suitable for clinical practice (75).

For IL-6, a recent clinical trial of blocking the effects of IL-6 by intravenous infusion of tocilizumab (humanised anti-IL-6R antibody) found that it reduced the number of PCI-treated peri-procedural myocardial injury in patients with NSTEMI, but whether this treatment is beneficial in patients with STEMI treated with PCI needs to be validated (76).

### 4.2 Targeting complement systems and adhesion molecules

For the complement system, clinical trials investigating pexelizumab (an anti-C5 complement antibody) did not find any clinical benefit in STEMI patients treated with PCI and CABG (77, 78). In addition, although intravenous C1 inhibitor treatment reduced myocardial injury in STEMI patients treated with CABG, the study sample size was small and the effect on STEMI patients treated with PCI needs to be validated (79). Interestingly, another study of 322 patients with NSTEMI found that treatment with Inclacumab (a recombinant P-selectin monoclonal antibody) before PCI successfully reduced myocardial injury. However, it is unclear whether this treatment is effective in STEMI patients undergoing PCI (80).

In summary, it is clear that immunotherapies developed for MI are currently immature.

## 5 Driving new immunomodulatory therapies by immune cells

It is important to think about the reasons for the failure of clinical translation for various inflammatory factors. First, there are some general issues here that are difficult to address: (I) Rodents such as mice have a larger and more resilient inflammatory response than other species (130), and applying results from animal experiments to humans will inevitably result in differences. (II) Current animal model preparations for MI still perform coronary artery ligation, and most patients with clinical MI have an atherosclerotic pathological process and may have comorbidities such as diabetes, hypertension, and obesity disease, so the models are not a perfect match. (III) Subjects taking concurrent drugs such as statins and beta-blockers also increase individual differences, which cannot be completely eliminated prior to the trial. (IV) Effective doses suitable for clinical application are difficult to obtain from preclinical studies, and the timing of dosing is very important for patients, and application at different times is likely to lead to widely varying results.

However, of relatively greater importance is that targeting various inflammatory factors or DAMPs may not be appropriate. This is because their effects may also be bidirectional but unarticulated and their effects are too broad in scope. In other words, these targets are not precise enough. Therefore, finding more precise targets to redesign targeting strategies may be more beneficial to achieve immunomodulatory therapies for MI.

As previously described, each of the immune cells infiltrating after MI plays an important role in ventricular remodeling, particularly monocytes/macrophages, throughout the process of ventricular remodeling. The fact that most immune cells are functionally bidirectional, while reflecting the difficulty of elucidating the post-MI immune process, also suggests that immune cells themselves have a large number of available targets. By finding and modulating more precise targets associated with immune cells, such as surface receptors or self-expressed functional proteins, leading to alterations in immune cell phenotype or function, or even by altering intercellular communication, the post-MI microenvironment may be improved, leading to a more modest ventricular remodeling outcome.

We believe that immune cells have the potential to become the protagonists of novel immunomodulatory therapies, as they allow for more precise targeted therapies. Although many potential targets have been proposed in animal experiments with various immune cells, the failure of previous clinical trials in suppressing neutrophils suggests that future research must focus on a balanced strategy to suppress the deleterious effects of inflammation while maintaining and promoting anti-inflammatory processes, and achieving balance also requires better precision in the first place.

## 6 Targeting strategies worth considering for novel immunomodulatory therapies

### 6.1 Direct targeting of antibodies

**Figure 3:**
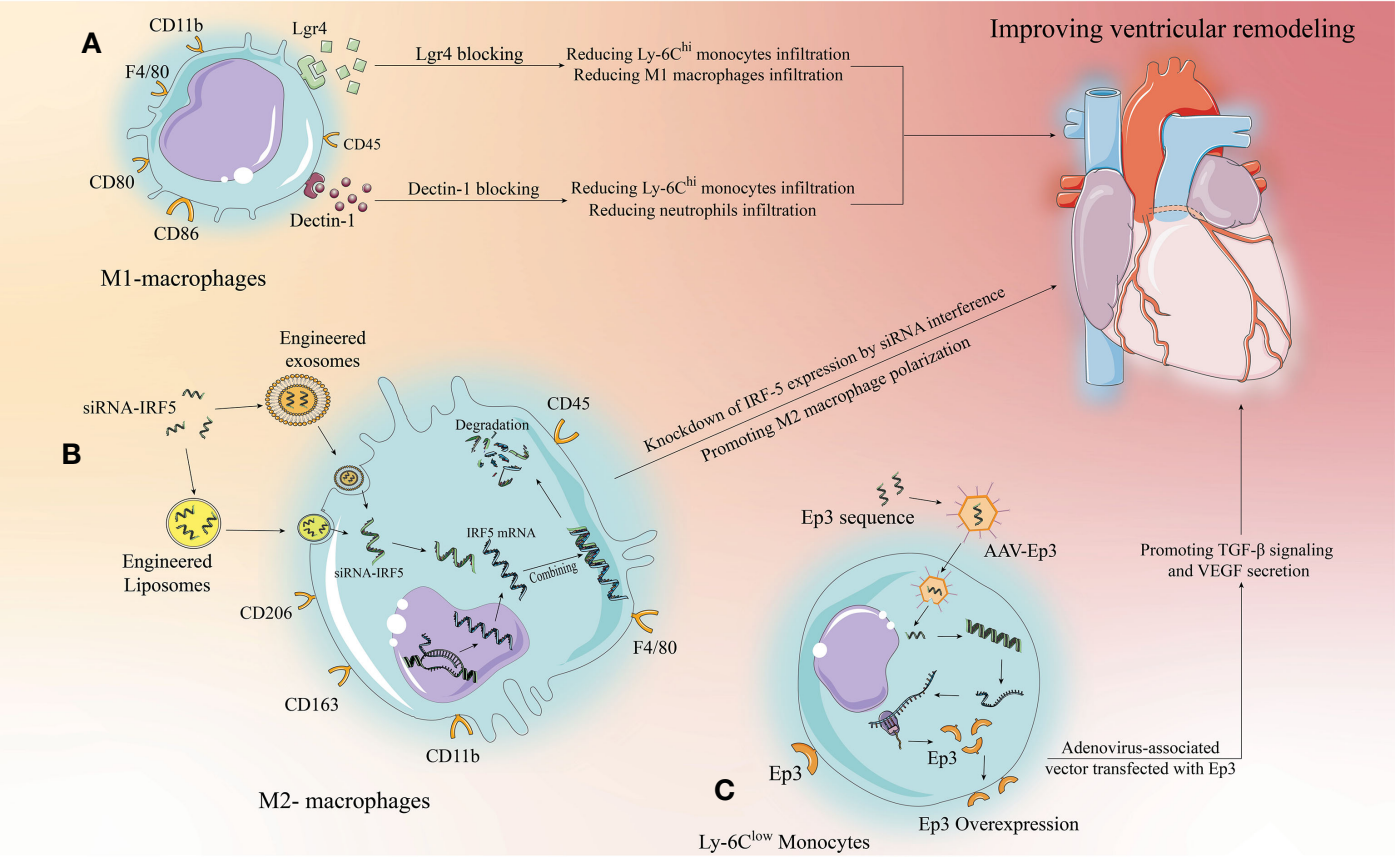
Targeting strategies for the development of new immunomodulatory therapies with immune cells. Monocytes/macrophages are used as an example for illustration, we propose three targeting strategies. **(A)** Extracellular specific blockade of surface receptors based on preclinical studies to modulate relevant signaling pathways to modify the inflammatory response and improve ventricular remodeling. **(B)** Liposomes and exosomes as drug carriers can effectively target macrophages and be phagocytosed by macrophages to release the drug. siRNAs designed to target and inhibit relevant target proteins can enter macrophages *via* liposomes or exosomes to knock down the expression of pro-inflammatory target proteins, thereby altering the inflammatory microenvironment to improve ventricular remodeling. **(C)** AAV has been developed rapidly in recent years, with low toxicity and strong targeting. Designing anti-inflammatory target protein sequences to be loaded by AAV to target and increase the expression of anti-inflammatory target proteins in target cells, thereby inhibiting inflammation and improving ventricular remodeling.

For surface receptors on immune cells that have been tapped in preclinical experiments, such as Lgr4 on the surface of macrophages as described above, monoclonal antibodies specific for Lgr4 can be designed to directly target and block the corresponding pathway. However, this approach may be limited to the extracellular and may have relatively more side effects due to the extensive nature of gene expression (**Figure 3A**).

### 6.2 Targeted knockdown of target protein expression by small interfering RNA-conjugated drug vectors

The development of drug carriers is of great significance for clinical translation, and exosomes and liposomes have shown great potential. First, they have a phospholipid bilayer surface that facilitates phagocytosis by target cells; second, they are structurally stable and have some targeting ability; more importantly, they are highly plastic and can be modified to enhance immune evasion and targeting, making them well suited as drug delivery vehicles (131, 132). Excitingly, recently, Li et al. demonstrated that platelet membrane-modified exosomes have a significant ability to target monocytes/macrophages that move with Ly-6C^hi^ monocytes to the cardiac ischemic zone after MI and are later phagocytosed by differentiated M1 macrophages, a study that provides important evidence for drug carrier targeting of immunomodulatory therapies (133). In the case of IRF-5 described above, siRNAs targeting IRF-5 were designed to be loaded into engineered and modified exosomes or liposomes to target and inhibit macrophage to express IRF-5, thereby promoting M2 macrophage polarization (**Figure 3B**). This approach has a great range of applications, but it relies on well-established drug delivery systems. Current research on exosome and liposome engineering is developing rapidly and reflects good promise for application, but it will take some time to develop a mature system.

### 6.3 Promoting target protein expression using adeno-associated viral vector carrying target protein sequences

The research on AAV is relatively the most mature, and relevant drugs have been applied clinically in the U.S (134).. AAV can carry single-stranded DNA sequences of target proteins, which do not integrate into chromosomes after entering the target cells, but can still exist independently in the cytoplasm and undergo prolonged transcription and translation to overexpress the target proteins in the target cells. Therefore, this targeting strategy is well suited to target cardiomyocytes, a class of cells with poor replication capacity, and have an important role in driving clinical translation of cardiomyocyte regeneration (134). Furthermore, similar to exosomes and liposomes, AAV itself has few side effects, high targeting capacity, and is malleable and can likewise be engineered to obtain higher or more unique targeting capacity (135). Thus using AAV for immunomodulation is a way to consider, for example, the aforementioned Ep3 receptor, where the single-stranded sequence of Ep3 is delivered to monocytes *via* AAV to promote Ep3 overexpression, thus better attenuating inflammation and promoting repair (**Figure 3C**).

## 7 Future perspectives

Achieving good translational medicine requires that good precision medicine be accomplished first. The wide range of roles played by various immune cells after MI and the targets that have been identified reflect their future therapeutic potential, and we believe it is only a matter of time before we continue to explore this path and discover more precise and effective targeting strategies to drive new immunomodulatory therapies into the clinic.

Based on the results available, future research directions can be focused on the following areas: (I) Combining transcriptomics and metabolomics to distinguish various immune cell subpopulations in more detail and assess differences in recruitment characteristics and function of different subpopulations to provide evidence for achieving more precise targeted therapies. (II) Based on gene sequencing technologies, analyze differentially expressed genes in post-MI immune cells, screen and continue to refine preclinical studies, paying particular attention to distinguishing whether these targets primarily regulate inflammatory or reparative responses, which is critical for assessing the timing of drug administration. (III) Mining the communication networks between immune cells after MI, such as extracellular vesicles that are extensively involved in the MI repair process, from which more potential targets can be explored, especially the various miRNAs delivered by exosomes. (IV) Targeting only pro-inflammatory targets may have limitations. This approach may not lead to significant alterations in other pathophysiological processes, particularly vascular regeneration and fibroblast activation during the repair phase. Assessing the feasibility of multi-target combinatorial targeting is also a direction worth considering.

Furthermore, in order to better facilitate the clinical translation of immunomodulation, we believe that the following aspects need to be refined: (I) Preclinical experiments using animals with higher clinical relevance to humans. Although it is difficult to mimic human MI secondary to atherosclerosis, we should ensure that the target under study adequately influences the immune cascade response following MI. (II) Dosing should be timed to coincide as much as possible with the functional properties of the target; for example, targets that act in a pro-inflammatory manner may be more appropriate for early use. (III) Patient heterogeneity in clinical trials cannot be completely avoided, but major influencing factors should be excluded as much as possible. Notably, due to the current widespread use of interventional techniques, ischemic time may also have an impact on trial results; patients with shorter ischemic time have less myocardial cell death and may benefit more from reperfusion, so care should be taken to control for the degree of initial preoperative injury in subjects, which may make the trial results more convincing. (IV) Patient genetic factors, co-morbidities, and age may have an impact on inflammation levels (136). Some patients exhibit more severe inflammation after infarction, and these patients may benefit from interventions targeting early inflammation control. Conversely, the main problem in certain patients after infarction is the development of significant myocardial hypertrophy and fibrosis, which is particularly common in patients with comorbid diabetes mellitus, and such patients may be better suited to interventions targeting fibrosis and remodeling during the repair phase (137). Therefore, it is worth thinking about the need to group patients according to different disease characteristics and to design more effective clinical trials or treatment protocols.

## 8 Conclusion

Ventricular remodeling after MI is the result of multiple factors, and immunomodulatory therapies have emerged based on the critical role of various inflammatory factors and immune cells in this process. However, the clinical trials we conducted did not produce effective results. Therefore, we believe that new immunotherapies should target more precise targets. Focusing on different immune cells and tapping into more precise targets, rather than simply and generically suppressing the inflammatory response by inhibiting a certain inflammatory factor, may improve the results of clinical trials. In conclusion, the field of immunomodulatory therapies for MI is still in its infancy, and the parallel development of drug delivery vehicles will drive clinical translation, while the use of immune cells to drive more precise therapies is expected to lead to new breakthroughs.

## Author contributions

WN identified the manuscript ideas and wrote most of the manuscript. ZH wrote parts of the manuscript, collected references and prepared figures. All authors contributed to the manuscript and approved the submitted version.
